# Cancer risk in patients with constitutional chromosome deletions: a nationwide British cohort study

**DOI:** 10.1038/sj.bjc.6604391

**Published:** 2008-05-27

**Authors:** A J Swerdlow, M J Schoemaker, C D Higgins, A F Wright, P A Jacobs

**Affiliations:** 1 Section of Epidemiology, Institute of Cancer Research, Sir Richard Doll Building, Cotswold Road, Sutton, Surrey SM2 5NG, UK; 2 Cell and Molecular Genetics Section, MRC Human Genetics Unit, Crewe Road, Edinburgh EH4 2XU, UK; 3 Wessex Regional Genetics Laboratory, Salisbury District Hospital, Salisbury, Wilts SP2 8BJ, UK

**Keywords:** constitutional chromosome deletions, cohort, risk

## Abstract

The finding of increased risks of specific cancers in individuals with constitutional deletions of chromosomes 11p and 13q led to the discovery of cancer predisposition genes at these locations, but there have been no systematic studies of cancer risks in patients with constitutional deletions, across the chromosome complement. Therefore, we assessed cancer incidence in comparison with national cancer incidence rates in a follow-up of 2561 patients with constitutional autosomal chromosome deletions diagnosed by microscopy or fluorescence *in situ* hybridisation in Britain during the period 1965–2002. Thirty cancers other than non-melanoma skin cancer occurred in the cohort (standardised incidence ratio (SIR)=2.4, 95% confidence interval (CI) 1.6–3.5). There were significantly increased risks of renal cancer in persons with 11p deletions (SIR=1869, 95% CI 751–3850; *P*=4 × 10^−21^), eye cancer with 13q deletions (SIR=1084, 95% CI 295–2775; *P*=2 × 10^−11^), and anogenital cancer with 11q deletions (SIR=305, 95% CI 63–890; *P*=3 × 10^−7^); all the three latter cancers were in the 11 subjects with 11q24 deletions. The results strongly suggest that in addition to suppressor genes relating to Wilms' tumour risk on 11p and retinoblastoma on 13q, there are suppressor genes around 11q24 that greatly affect anogenital cancer risk.

Visible constitutional chromosome deletions are present in about 0.5–1 in 10 000 newborn babies (Hamerton *et al*, 1975; Jacobs *et al*, 1992). Such deletions can give information in a unique way about the function and consequences of the genes on the parts of the chromosome deleted. Thus, if tumour suppressor genes play an important role in the risk of a type of cancer, it might be expected that the risk of that cancer would be increased in individuals who have deletions that include the relevant gene. The cells of most malignancies show chromosome abnormalities, many of which are specific to a particular tumour type(s), frequently the loss of a specific chromosomal band or segment (Yunis, 1983; Gasparini *et al*, 2007); deletion of tumour suppressor genes is increasingly regarded as a key initiating event in epithelial tumours (Gasparini *et al*, 2007). Increased risks of certain cancers in patients with constitutional chromosome deletions have led to the identification of cancer loci – 13q14 deletions for retinoblastoma and 11p13 deletions for Wilms' tumour – and it is also important to know about them for clinical care and surveillance and for giving advice to patients and their relatives.

No studies, however, appear to have investigated systematically cancer risks in patients with deletions of each chromosome and arm. A Danish cohort study (Bache *et al*, 2006) investigated cancer incidence risk in patients with deletions overall but did not divide the analysis by chromosome, and a US cohort study analysed cancer incidence risks in the first 4 years of life in patients with Beckwith–Wiedemann syndrome (DeBaun and Tucker, 1998). Several studies have investigated the frequency of chromosome abnormalities in patients with haematological malignancy (Benitez *et al*, 1987; Cerretini *et al*, 2002; Welborn, 2004), but with far too few deletions to assess whether risk is altered for deletions on specific chromosomes.

Therefore, we undertook a national cohort study of cancer incidence in patients with chromosome deletions diagnosed by light microscopy or fluorescence *in situ* hybridisation (FISH) in Britain during the past 40 years.

## MATERIALS AND METHODS

From all 27 cytogenetic laboratories in Britain except two small laboratories, we extracted information about all live-born patients diagnosed with autosomal chromosome deletions detectable on light microscopy or by FISH since the laboratories opened or from as long ago as records had been maintained. Ethical approval was obtained from the relevant ethics committees. We excluded from the cohort patients whose cytogenetic records showed that they had been karyotyped because of cancer and also patients with a deletion plus trisomy, because the latter may be related to cancer risk in its own right.

Information regarding identification of the cohort members was sent to the National Health Service Central Registers (NHSCRs) for England and Wales and for Scotland. These registries had records of all NHS patients of their respective countries and, consequently, are virtually complete population registers; they record deaths, emigrations, other exits from follow-up and, since 1971, cancer registrations. The cohort members were ‘flagged’ on the registers to obtain information about cancer incidence, deaths, and other follow-up. The sites of cancers were coded according to the revision of the International Classification of Diseases (ICD) (World Health Organization, 1977) in force in Britain at the time of incidence: ICD8 from 1971 to 78, ICD9 from 1979 to 94 in England and Wales and from 1979 to 96 in Scotland, and ICD10 from 1995 onwards in England and Wales and from 1997 onwards in Scotland. We then bridge coded the cancer data (i.e. matched equivalent codes between different ICD revisions) to produce the ICD9 categories shown in the tables.

To analyse cancer incidence risks in the cohort, we calculated person-years of follow-up by sex, 5-year age group, calendar year and country (England and Wales *vs* Scotland), beginning from the date of cytogenetic diagnosis or 1 January 1971, whichever was latest, and ending on 31 December 2004 (the date up to which national cancer registration data were reasonably complete at the time of analysis) or the date of death, emigration, other loss to follow-up or 85th birthday, whichever occurred first. Non-melanoma skin cancer was excluded from analysis because its registration was seriously incomplete (Swerdlow *et al*, 2001). Follow-up was censored at age 85 because at older ages cancer diagnosis is likely to be incomplete and inaccurate, and national (i.e. expected) cancer incidence rates are not available by 5-year age group. We then computed expected site-specific cancer incidence in the cohort by multiplying age-, sex-, calendar year- and country-specific person-years at risk in the cohort by the corresponding national cancer incidence rates. Standardised incidence ratios (SIRs) were calculated as the ratio of observed to expected deaths, and 95% confidence intervals (CIs) for the SIRs were calculated based on Fisher's exact method (Breslow and Day, 1987). All significance tests were two-sided. To assess the significance of the results allowing for the fact that multiple testing had been undertaken, we used the Bonferroni adjustment (Altman, 1991).

To assess whether the apparent increase in cancer incidence might have been caused by selective cytogenetic diagnosis of persons ill with as yet undiagnosed cancer, or by imprecision in the exact dates of cytogenetic and cancer diagnoses when these were close together, we examined the duration between cytogenetic and cancer diagnosis for the cancers incident during cohort follow-up, and we reanalysed risks omitting follow-up and cancers occurring in the year after cytogenetic diagnosis.

## RESULTS

From the records of the cytogenetic laboratories included in the study, we identified 3212 patients with autosomal chromosome deletions diagnosed during 1965–2002. In most of the laboratories, cytogenetic records were available back to the 1960s or early 1970s, depending on the date when the laboratory was founded and how far back records had been retained. Fifteen patients were excluded from the study because their cytogenetic testing was undertaken as a consequence of a cancer diagnosis (5 patients with 13q deletions and retinoblastoma, 4 with 11p deletions and Wilms' tumour), 68 patients because the year of karyotyping was unknown, 517 cases because they could not be flagged, generally because the exact date of birth or full name was unknown and 22 patients were excluded for other reasons. There remained 2561 subjects who were flagged at the NHSCRs and who comprised the study cohort. Twenty-four of these subjects had been identified from the research-based register of the MRC Human Genetics Unit and the other 2537 from registers of clinical diagnostic laboratories.

The patients included in the cohort were mainly diagnosed at ages under 15 years (79%) and there were slightly more female patients than male patients (52 *vs* 48%, respectively); most were diagnosed in 1990 or later (81%) and only 3% before 1980 (Table 1). The most common deletions were those of 22q, 15q, 7q, 5p and 17p. The 651 subjects with autosomal deletions who were diagnosed at the study centres but not flagged and not included in the cohort (not in table) had a similar sex and age distribution to the cohort members except that they included a somewhat greater proportion of infants and they included all subjects of unknown age (i.e. those with an unknown date of birth, which made flagging impossible and therefore led to exclusion from the cohort).

A total of 252 subjects died during follow-up, 42 emigrated or were otherwise lost to follow-up and 2267 survived to the end of follow-up or to age 85. The cohort members were followed-up for a total of 27 386 person-years, an average of 10.5 years per subject. Thirty-two cancers were recorded as occurring in the cohort (Table 2), of which two were non-melanoma skin cancers and were excluded from analysis. Cancer risk was significantly increased in the cohort (SIR=2.4, 95% CI 1.6–3.5), largely because of greatly increased risks of renal, eye and female genital cancers and of leukaemia. The coding of cancers in the national data (i.e. the ‘expected’ rates for this study) does not enable analysis of risks of Wilms' tumour and retinoblastoma *per se*, but all of the renal and eye cancers occurring in the cohort were these two tumours, respectively. In total, five anogenital cancers occurred in the cohort (SIR=8.5, 95% CI 2.7–19.7): two vulval, one vaginal, one anal and one cervical.

When we analysed cancer risks by the chromosome and arm of deletion, all 8 renal cancers were in the 50 patients with 11p deletions, all 4 eye cancers were in the 82 patients with 13q deletions and there were 3 anogenital cancers (2 vulval, 1 anal) in the 36 patients with 11q deletions. Table 3 shows risks of these tumours by sex and age. Renal cancer risk in subjects with 11p deletions was almost 2000-fold increased (SIR=1869, 95% CI 751–3850; *P*=4 × 10^−21^), and was comparably increased in male and female subjects; all cases occurred at ages under 5 years, for which the SIR was almost 4000. The risk of eye cancer in subjects with 13q deletions was 1084 (*P*=2 × 10^−11^). The relative risk of vulval and vaginal cancer in patients with 11q deletions was 2930 (*P*=5 × 10^−7^) and of the wider category of anogenital cancers it was 305 (*P*=3 × 10^−7^). All of these SIRs remained highly significant (*P*<0.001) after application of a Bonferroni adjustment.

The renal cancers all occurred in patients with deletions that encompassed 11p13, except one with a break at 11p14. The eye cancers were all in patients with deletions encompassing 13q14 except that for one the breakpoint was not specified. The two vulval cancers and the anal cancer in patients with deletions of 11q all occurred in subjects with deletions of 11q24 and were all of squamous cell histology. The vulval cancers occurred at ages 22 and 36 years and the anal cancer at age 46 years; the breakpoints for the vulval cancer patients were recorded as 11q24.2; for the anal cancer, no further precision beyond 11q24 was recorded. The vaginal cancer was an adenocarcinoma and occurred in a patient with a deletion of 22q11; the cervical cancer was a squamous cell cancer in a patient with a 15q deletion. There were 36 subjects in the cohort with 11q deletions, of whom 11 were known to have an 11q24 breakpoint, 21 to have deletions with breakpoint(s) elsewhere on the arm and 4 with deletions of unknown breakpoint(s).

The four cases of leukaemia occurred in patients with different deletions: one ALL in a patient with an 8p deletion, one AML in a patient with a 20q deletion, one acute leukaemia NOS in a patient with a deletion on chromosome 5 and one CML in a patient with an 18q deletion. Likewise, the two testicular cancers were heterogeneous: one in a man with a 15q deletion and the other in a man with an 18q deletion.

All but one of the eight renal cancers (Wilms' tumours) occurred at least a year after cytogenetic diagnosis of a constitutional deletion, as did all of the anogenital cancers (indeed, the earliest of these was 7 years after cytogenetic diagnosis). Two of the four leukaemias and all of the eye cancers (retinoblastomas) were recorded as occurring within a year after the cytogenetic diagnosis, but the eye cancers all occurred at ages under 6 months, so only periods shorter than this between the two diagnoses were possible.

When risks were reanalysed excluding the first year of follow-up, the risk of cancer (excluding non-melanoma skin cancer) overall in the cohort remained significantly increased (SIR=1.9, 95% CI 1.2–2.9) and of leukaemia was increased but not significantly (SIR=2.2, 95% CI 0.3–7.9); the SIRs for renal cancer in patients with an 11p deletion and anogenital cancer in patients with an 11q deletion were greatly increased and highly significant, and there were no cases of eye cancer occurring beyond 1 year of follow-up in subjects with 13q deletions.

## DISCUSSION

In this national cohort, we found greatly increased risks of retinoblastoma, Wilms' tumour and anogenital cancer in relation to deletions of particular chromosome arms. There appear to be no previous such cohort data with which to compare these results. Two methodological aspects of the study need consideration, although they seem unlikely to explain the results. First, subjects were omitted from the cohort if their identifying information from the cytogenetic centre was too incomplete to allow ‘flagging’ at the NHSCR, or if they were from early years of records not retained by the cytogenetic centre: these omissions, however, relate to general record-keeping of cytogenetic testing, not subsequent cancer or follow-up, and therefore are very unlikely to have biased our results. Second, not all patients with deletions in the country will necessarily have reached cytogenetic diagnosis. From the prevalence of microscopically visible deletions at birth (0.5–1.0 per 10 000) (Hamerton *et al*, 1975; Jacobs *et al*, 1992), we estimate that all or almost all cases born nationally in the early and middle 1990s were within our data set, but that there was underdiagnosis for earlier periods. It is difficult to assess completeness of diagnosis by FISH because the probes enabling FISH diagnosis came to be generally used at different dates for different microdeletions. Again, however, there is no reason to believe that underdiagnosis would be related to future cancer risk, other than if a cancer diagnosis, or prediagnostic symptoms of cancer, itself led to cytogenetic testing. Therefore, we excluded from the cohort patients known to have been karyotyped because of cancer and examined the effect of excluding from analysis events and follow-up in the year after cytogenetic testing.

Deletions of 11p in Wilms' tumour cells and 13q in retinoblastoma cells in sporadic as well as constitutional cases (the latter having the deletion in all cells of the body) strongly suggest that the deletion is important to the aetiology of these tumours, and that when present constitutionally it is the reason for greatly increased risk (Yunis, 1983). This has led to the discovery of the RB and WT1 genes. Many other deletions have been found in human malignancies (Mitelman *et al*, 1997), but none are known to affect cancer risk when present constitutionally.

Our study enabled quantification of the well-established risks of Wilms' tumour and retinoblastoma in patients with 11p and 13q deletions, respectively, but the increased risk that we found for cancer of the vulva (or more broadly anogenital cancers) in patients with 11q24 deletions, however, has no precedent and hence must be interpreted with caution. On the one hand, we examined risks for a large number of cancer sites for a large number of different deletions; hence, some significant results would be expected by chance alone. On the other hand, the *P*-value for the risk was very extreme (3 × 10^−7^) and remained highly significant after Bonferroni adjustment (i.e. after allowing for multiple testing), and there is considerable plausibility to the finding of increased cancer risk: losses in 11q13–23 are often found in squamous cell cancers of the vulva and vagina (Micci *et al*, 2003), and 11q23–ter deletions in anal cancers (Muleris *et al*, 1987), as well as deletions in this area in several other cancers (Mitelman *et al*, 1997). Loss of heterozygosity, indicating potential presence of a tumour suppressor gene, has been seen frequently at 11q13–22 in squamous cell vulval cancers (Pinto *et al*, 1999) and at 11q23 in cervical cancers (Hampton *et al*, 1994; Skomedal *et al*, 1999; Pulido *et al*, 2000; O′Sullivan *et al*, 2001). Cancers of the vulva, cervix, anus and penis have very closely related aetiology from sexually transmitted viruses, so it is reasonable to group them together when seeking aetiological mechanisms and predispositions. As our cancer information comes from cancer registrations, we cannot determine whether the particular cancers in our 11q24 deletion patients contained HPV DNA.

Patients with 11q terminal deletion disorder (Jacobsen's syndrome) usually have a breakpoint at 11q23.3, with a deletion extending to the telomere. This breakpoint has been shown to map within the same 100 kb interval as the fragile site FRA11B, which includes part of the CBL2 oncogene (Jones *et al*, 1994). The tumour suppressor genes CHEK1, BARX2 and OPCML are often deleted in individuals with 11q terminal deletions (Grossfeld *et al*, 2004). One of the largest human genes, DKFZp686H, is located at 11q25, close to a common fragile site – an area of profound genomic instability (Smith *et al*, 2006). Chromosomal bands 11q24–25 contain over 100 genes (UCSC Genome Browser, 2006). These include a number of known or potential tumour suppressor genes (BCSC-1, CHEK1, ST14, ATM, P53AIP1), genes with proposed roles in cancer progression (BARX2), apoptosis (PIG8, P53AIP1) and oncogenesis (FLI1, ETS1), and a DNA damage-inducible gene (DDI1). Further work will be required to clarify whether deletion of any of these genes is involved in the apparent excess of anogenital cancers.

The only other significant finding in the study was an increased risk of leukaemia in the cohort overall. This was only just significant, however, with two cases recorded as having cancer diagnosis close to the date of cytogenetic diagnosis, and each of the four cases having a deletion on a different chromosome, so the finding does not provide any substantial evidence for an aetiological relationship.

In conclusion, follow-up of patients with constitutional chromosome deletions has shown highly significant, greatly increased, specific risks of three cancers. For two, renal cancer in patients with 11p deletions and eye cancer in patients with 13q deletions, this enabled quantification of known risks; the third, anogenital cancer in patients with 11q (terminal) deletions, is a previously unreported high risk that needs further investigation of potential predisposition genes at this location.

## Figures and Tables

**Table 1:** Cohort by sex, age at diagnosis, and chromosome and arm of deletion

	**Male**	**Female**	**Total**
	**No.**	**No.**	**No.**
*Age at diagnosis (years)*			
<1	413	422	835
1–14	591	595	1186
15–24	98	128	226
⩾25	123	191	314
			
*Year of diagnosis*			
<1980	20	49	69
1980–89	189	229	418
⩾1990	1016	1058	2074
			
*Chromosome and arm of deletion*			
1p	9	13	22
1q	12	15	27
2p	7	4	11
2q	44	51	95
3p	6	6	12
3q	7	12	19
4p	37	47	84
4q	34	40	74
5p	57	83	140
5q	13	16	29
6p	8	6	14
6q	22	13	35
7p	5	4	9
7q	108	102	210
8p	18	19	37
8q	10	6	16
9p	23	34	57
9q	10	7	17
10p	7	3	10
10q	23	29	52
11p	24	26	50
11q	14	22	36
12p	4	5	9
12q	3	4	7
13q	37	45	82
14q	8	8	16
15q	237	223	460
16p	4	3	7
16q	5	7	12
17p	61	62	123
17q	6	0	6
18p	27	32	59
18q	48	69	117
19p	3	0	3
19q	0	0	0
20p	4	8	12
20q	2	0	2
21q	11	10	21
22q	261	283	544
Not known^a^	6	19	25
			
Total	1225	1336	2561

a Either deletion of known autosome but unknown arm or deletion of known chromosome group (e.g. C) but unknown specific chromosome.

**Table 2:** Cancer incidence risks in the overall cohort, by site

**ICD9 code**	**Cancer site**	**No. of cancers**	**SIR**	**95% CI**
150	Oesophagus	1	6.6	0.2–36.7
151	Stomach	1	4.4	0.1–24.3
153, 154	Colon+rectum	1	1.2	0.0–6.7
155	Liver	1	11.7	0.3–65.4
157	Pancreas	0	0	0–24.9
162	Lung	0	0	0–4.0
174, 175	Breast	1	0.4	0.0–2.5
180	Cervix	1	2.0	0.1–11.2
183	Ovary	0	0	0–8.9
184	Other female genital organs	3	59.9	12.4–175.2^a^
185	Prostate	0	0	0–10.1
186	Testis	2	4.8	0.6–17.2
188	Bladder	1	3.5	0.1–19.3
189	Kidney	8	27.5	11.9–54.1^a^
190	Eye	4	48.3	13.2–123.7^a^
191–192, 225, 237.5, 237.6, 237.9, 239.6	Nervous system	1^b^	1.0	0–5.5
200, 202	Non-Hodgkin's lymphoma	0	0	0–6.1
201	Hodgkin's disease	1	2.4	0.1–13.2
204–208	Leukaemia	4	3.8	1.0–9.8^c^
140–172, 174–208	All malignancies except non-melanoma skin cancer	30	2.4	1.6–3.5^a^

ICD=International Classification of Diseases; SIR=standardised incidence ratio; CI=confidence interval.

a *P*<0.001.

b One meningioma, not included in ‘all malignancies’. In addition, two non-melanoma skin cancers were recorded.

c *P*<0.05.

**Table 3:** Risks of selected cancers in patients with selected deletions, by sex and attained age

**Chromosome and arm of deletion, cancer site**	**Sex and age (years)**	**No.**	**SIR**	**95% CI**
11p, renal cancer	Male	4	2220	605–5683^a^
	Female	3	1544	318–4511^a^
	0–4	7	3197	1285–6587^a^
	5–14	0	0	0–21 311
	⩾15	0	0	0–12 632
	All ages, both sexes	7	1869	751–3850^a^
				
13q, eye cancer	Male	0	0	0–1781
	Female	4	2469	673–6321^a^
	0–4	4	2023	551–5180^a^
	5–14	0	0	0–9225
	⩾15	0	0	0–2807
	All ages, both sexes	4	1084	295–2775^a^
				
				
11q, vulval and vaginal cancer		2	2930	355–10 586^a^
				
11q, anogenital cancers^b^	Male	0	0	0–17 181
	Female	3	311	64–910^a^
	0–14	0	0	0–28 540
	15–44	2	230	28–831^a^
	⩾45	1	978	25–5447^c^
	All ages, both sexes	3	305	63–890^a^

SIR=standardised incidence ratio; CI=confidence interval.

a *P*<0.001.

b Cervix, vulva, vagina, anus, penis.

c *P*<0.01.

## Appendix

The group comprises, in addition to the above-mentioned authors, Paul J Batstone (Inverness Genetics Laboratory), Leslie J Butler (NE Thames, Great Ormond Street Hospital Genetics Laboratory), Teresa Davies (Bristol Genetics Laboratory), Valerie Davison (Birmingham Genetics Laboratory), Zoe Docherty (SE Thames, Guy's Hospital Genetics Laboratory), David P Duckett (Leicestershire Genetics Centre), Margaret Fitchett (Oxford Genetics Laboratory), Alison Fordyce (MRC Human Genetics Unit, Edinburgh), Lorraine Gaunt (Manchester Genetics Laboratory), Elizabeth Grace (Edinburgh Genetics Laboratory), Peter Howard (Liverpool Genetics Laboratory), Gordon W Lowther (Glasgow Genetics Laboratory), Christine Maliszewska (Dundee Genetics Laboratory), Edna L Maltby (Sheffield Genetics Laboratory), Kevin P Ocraft (Nottingham Genetics Laboratory); Selwyn Roberts (Wales Genetics Laboratory), Kim K Smith (Cambridge Genetics Laboratory), Gordon S Stephen (Aberdeen Genetics Laboratory), John W Taylor (SW Thames, St George's Hospital Genetics Laboratory), Catherine S Waters (NW Thames, Northwick Park Hospital Genetics Laboratory), Jeffery Williams (Leeds Genetics Laboratory), John Wolstenholme (Newcastle Genetics Laboratory), and Sheila Youings (Wessex Regional Genetics Laboratory).
